# Long Noncoding RNA (lncRNA)-Mediated Competing Endogenous RNA Networks Provide Novel Potential Biomarkers and Therapeutic Targets for Colorectal Cancer

**DOI:** 10.3390/ijms20225758

**Published:** 2019-11-16

**Authors:** Liye Wang, Kwang Bog Cho, Yan Li, Gabriel Tao, Zuoxu Xie, Bin Guo

**Affiliations:** Department of Pharmacological and Pharmaceutical Sciences, College of Pharmacy, University of Houston, Houston, TX,77204, USA; kcho7@central.uh.edu (K.B.C.); yanli93776@gmail.com (Y.L.); gtao@central.uh.edu (G.T.); zuoxu.xie@gmail.com (Z.X.)

**Keywords:** colorectal cancer, lncRNA, miRNA, ceRNA

## Abstract

Colorectal cancer (CRC) is the third most common cancer and has a high metastasis and reoccurrence rate. Long noncoding RNAs (lncRNAs) play an important role in CRC growth and metastasis. Recent studies revealed that lncRNAs participate in CRC progression by coordinating with microRNAs (miRNAs) and protein-coding mRNAs. LncRNAs function as competitive endogenous RNAs (ceRNAs) by competitively occupying the shared binding sequences of miRNAs, thus sequestering the miRNAs and changing the expression of their downstream target genes. Such ceRNA networks formed by lncRNA/miRNA/mRNA interactions have been found in a broad spectrum of biological processes in CRC, including liver metastasis, epithelial to mesenchymal transition (EMT), inflammation formation, and chemo-/radioresistance. In this review, we summarize typical paradigms of lncRNA-associated ceRNA networks, which are involved in the underlying molecular mechanisms of CRC initiation and progression. We comprehensively discuss the competitive crosstalk among RNA transcripts and the novel targets for CRC prognosis and therapy.

## 1. Introduction

Colorectal cancer (CRC) is the third most commonly diagnosed and fatal malignancy, with nearly 1.8 million new cases and almost 861,000 deaths in 2018 around the world [1]. In the past decade, the diagnosis and treatment of CRC have been greatly improved. However, distant metastasis, especially liver metastasis, and the cell stemness properties of cancer cells lead to the poor prognosis and high fatality rate in CRC patients [2]. Recent studies on the biology of noncoding RNAs in CRC and new gene therapy strategies for delivering therapeutic RNA, such as noncoding RNA or small interfering RNA (siRNA), have drawn intense interest in the investigation of long noncoding RNAs (lncRNAs) in the molecular mechanisms of CRC [3,4,5,6,7,8]. Besides the characterized lncRNAs or microRNAs (miRNAs) in important CRC-related signaling pathways, such as Wnt/β-catenin, studies in recent years have identified a novel competitive RNA network including lncRNA, miRNA, and mRNA, which mediates CRC cell proliferation, invasion, tumor growth, and metastasis. Moreover, competitive endogenous RNA (ceRNA) crosstalk has strong relevance to the acquisition of chemo-/radioresistance in CRC. Hence, deeper exploration and understanding of ceRNA mechanisms in CRC development are needed.

## 2. LncRNA in CRC

**Figure 1:**
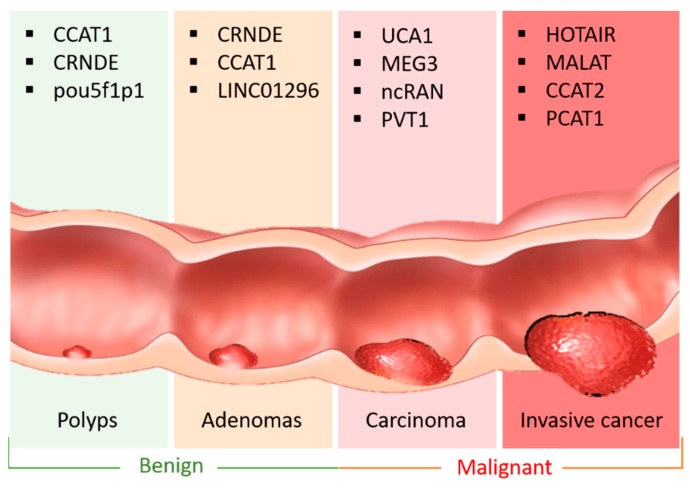
Representative lncRNAs in the different stages of CRC. There are four major stages of CRC development: precancerous polys, Adenomas, Carcinoma and invasive cancer. Representative lncRNAs involved in the certain stages could be regarded as early-stage diagnostic biomarkers to evaluate CRC progression or therapeutic targets to suppress CRC metastasis.

LncRNAs, defined as transcripts longer than 200 nucleotides, are not translated into proteins [9]. LncRNAs, aberrantly expressed in various types of cancer cells, play a vital role in several common hallmarks of cancer [10]. In general, lncRNAs exert regulatory functions at different levels of gene expression, including chromatin modification, transcription, and post-transcription. As for chromatin modification, lncRNAs interact with chromatin remodeling complexes to induce heterochromatin formation in specific genomic loci, resulting in reduced gene expression. Moreover, lncRNAs modulate transcription by interacting with RNA-binding proteins and co-activators of transcriptional factors, or by regulating the major promoters of their target genes. Mechanically, lncRNAs exert their regulatory effects through communication with DNA, mRNAs, ncRNAs, and proteins, serving as different functional molecules, such as signals, decoys, scaffolds, and guides in various cancer-related cellular processes [11,12]. Previous studies reported that lncRNAs work mainly as signaling molecules in many important CRC-related pathways, or as scaffold guides to decoy CRC-specific protein partners, as well as a *cis-* and *trans-* regulator element for gene expression. For example, the oncogenic lncRNAs mapped to chromosome 8q.24, such as CACS11, CCAT family, and PVT1, promote CRC progression by interacting with proteins to stimulate *myc* or other Wnt target gene expression at the posttranslational level [13]. Furthermore, lncRNAs (Figure 1) are frequently involved in different stages of CRC from precancerous polyps to distant metastasis, which could be considered potent diagnostic biomarkers [14,15]. In recent years, increasing studies have demonstrated the existence of lncRNA-mediated competitive RNA crosstalk in CRC progression (Table 1).

## 3. The ceRNA Hypothesis in Cancers

**Figure 2:**
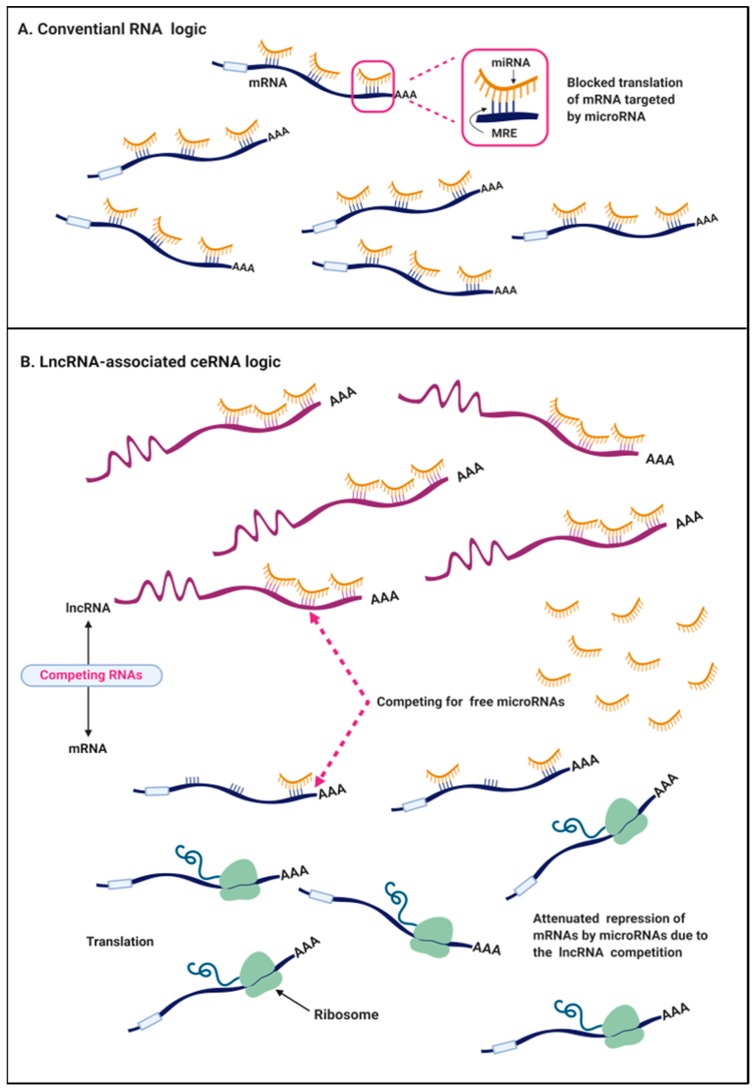
The competitive endogenous RNA (ceRNA) mechanism. (**A**) In the conventional crosstalk of RNA transcripts, in the cytoplasm, miRNAs exert the suppressive function on protein-coding mRNAs by base pairing with partial complementarity via the miRNA recognition element (MREs) mapped to the 3′UTR of mRNAs. (**B**) Under the ceRNA mechanism in cancer cells, aberrantly expressed long noncoding RNAs (lncRNAs) with MREs competitively sequestrate miRNAs and reduce the interaction between miRNA and mRNA, and thus attenuate the repression on the downstream mRNAs.

The conventional view in the RNA field is that microRNAs (miRNAs), the short endogenous RNA ~23 nt in length, pair to the miRNA recognition elements (MREs) in the protein-coding mRNAs by partial complementarity (Figure 2A) [16]. MREs are often located in 5′ untranslated regions (5′UTRs), coding sequences (CDS), and especially in 3′ untranslated regions (3′UTRs) of various types of RNA transcripts, such as ncRNA and mRNA. Once mature miRNAs guide the miRNA-programmed RNA-induced silencing complex (miR-RISC) to MREs, the mRNA destabilization or posttranslational repression will be actively triggered, leading to the inhibition of gene expression [17]. Most miRNAs pair imperfectly with their cognate mRNAs. A target prediction algorithm has showed that the pairing of miRNA nucleotides 2-8mer to the 3′UTR of the target mRNA is often important [16]. Each miRNA is able to target numerous MREs and repress up to hundreds of transcripts. The best estimate, considering all the 2-8mer perfect and imperfect 3′UTR matches, is that over 60% of human protein-coding genes are potential targets of miRNA, highlighting the involvement of miRNA‒mRNA interaction in diseases including cancer [18,19]. However, surprisingly, many cases of miRNA-mediated regulation are modest, which repress targeted protein expression by less than 2-fold, and usually yield no consequence at a physiological level [20,21]. This discrepancy hints that a large member of RNA communication has been missing, which likely contributes to the repressive effect of miRNA. 

**Figure 3:**
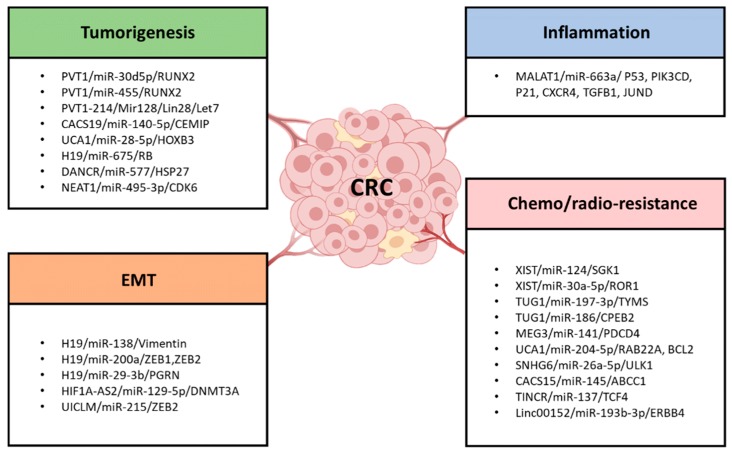
The lncRNA-associated ceRNA networks affect the four common hallmarks of colorectal cancer. Representative lncRNA‒miRNA‒mRNA networks are listed, which highlighted the involvement of lncRNA-ceRNA networks in four major hallmarks of CRC: tumorigenesis, EMT formation, inflammatory process and chemo-/radioresistance.

Since miRNA repressors began to be found in plants, animals, and artificial constructs, in 2009, Seitz proposed that a large proportion of RNA transcripts with MREs act as competitive inhibitors of miRNA, so-called “miRNA sponge,” which modulate miRNA expression and function by competing for miRNA binding sites with endogenous mRNAs [20]. Experimental observations supported his theory that the noncoding 3′UTR fragment inhibited miR-199a-3p expression and its activity by acting as a sponge of miRNA [22]. Notably, the competitive endogenous RNA (ceRNA) hypothesis was proposed by Selmena et al. in 2011 [19]. They speculated that ncRNAs, particularly lncRNAs, compete for the miRNA binding sites through partial complementarity, and they named these ncRNAs ceRNAs. This interplay leads to a decrease in the miRNA level and impairment of miRNA activity. The novel ceRNA logic has been validated in *Herpesvirus saimiri* noncoding RNA, which manipulates host-cell gene expression by degrading mature miR-27 in a binding-dependent manner [23]. Additionally, the effectiveness of the ceRNA pathway is primarily based on the relative levels of ceRNA transcripts. Changes at the ceRNA level are critical to potentiate or attenuate the functions of miRNA on target genes because of the intensified competition. In cancer cells and tissues, miRNAs might be more susceptible to degradation due to the interaction with the aberrantly expressed ncRNAs, especially lncRNAs, thereby regulating important cancer-related genes’ expression. Soon after the novel ceRNA notion was proposed, increasing bioinformatics data have identified that most cancer-related lncRNAs and protein-coding genes in the human genome densely contain MREs, which validates the existence of lncRNA‒miRNA‒mRNA logic in cancers (Figure 2B). Wang et al. first identified the lncRNA-associated ceRNA mechanism in liver cancer, where lncRNA HULC, as a sponge of miR-372, inhibited its activities and consequently reduced the repression of PRKACB [24]. Further experimental evidence indicated that the lncRNA-mediated ceRNA network plays a key role in the carcinogenesis of various cancers, including colorectal cancer, breast cancer, and ovarian cancer. Here, we specially discuss the recent identified lncRNA/ceRNA mechanisms in several hallmarks of colorectal cancer (Figure 3).

## 4. LncRNAs as ceRNA in CRC Tumorigenesis and Progression

Recent studies showed that lncRNA-related ceRNA crosstalk is closely related to the CRC initiation and progression. In this section, we discuss some ceRNA networks and their role in CRC cell proliferation, invasion, and metastasis. Also, we highlight the ceRNA regulatory networks consisting of a lncRNA/miRNA/mRNA axis.

### 4.1. PVT1/miR-30d-5p/RUNX2

LncRNA PVT1 is located at chromosome 8q.24.21, a region containing many CRC-associated lncRNAs, such as those involved in the Wnt signaling pathway [25]. PVT1 is highly upregulated in CRC cells and tissues. In CRC patients, upregulated PVT1 positively correlates with cell proliferation, invasion, tumor stages, and lymph node metastasis [15]. Previous studies reported that PVT1 promotes CRC development through its regulatory effect on c-myc protein [25]. The latest study conducted by Yu et al. revealed that PVT1 functions as a ceRNA in CRC via the PVT1/miR-30d-5p/RUNX2 axis [26]. Overexpressed PVT1 binds to miR-30d-5p directly, and such competitive binding decreases the abundance of miR-30d-5p and relieves its repression of the downstream target, RUNX2. RUNX2, a novel oncogene correlated with tumor growth and metastasis, can be regulated by several noncoding RNAs by an unclear mechanism [27,28,29,30]. This study presented a positive correlation of PVT1 and RUX2 in CRC tumor tissues in that PVT1 increased RUX2 expression. Likewise, supplementary studies showed that the RUX2 level was reduced in PVT1-knockdown CRC cell lines, but was induced in miR-30d-5p-knockdown CRC cells, suggesting that PVT1 promoted CRC tumorigenesis partly via this PVT1/miR-30d-5p/RUX2 ceRNA network.

### 4.2. PVT1/miR-455/RUNX2

Chai et al. reported that PVT1 served as a ceRNA of RUNX2 in CRC via the PVT1-miR-455-RUNX2 axis [31]. In CRC cells, PVT1 sponged miR-455 and negatively regulated miR-455 expression, which functions as a tumor suppressor in human cancers. The proliferation of CRC cells is markedly inhibited by PVT1 knockdown, along with increased miR-455, which could be rescued by the miR-455 inhibitor, suggesting that PVT1-induced CRC progression is partly caused by the inhibition of miR-455. Software predicted the potential binding sequences in miR-455 and 3′UTR of PVT1 and RUNX2. Subsequent experiments verified the competitive binding event and indicated that PVT1 promoted CRC development by sponging and inhibiting miR-455 to elevate RUNX2 expression.

### 4.3. PVT1-214/Mir128/Lin28/Let7

PVT1-214, one of the PVT1 transcripts, is upregulated in human CRC tissues. Recent research demonstrated that, in CRC cells, upregulated PVT1-214 stabilized the expression of Lin28 by harboring miR-128, which eventually repressed the Let7 family, the downstream target of Lin28. This study clearly showed that PVT1-214, as an oncogenic driver of CRC, promoted CRC tumorigenesis via the PVT1-214/miR-128/Lin28/Let7 axis [32].

Taken together, these findings indicated that lncRNA PVT1 acts as ceRNAs of several miRNAs. The identified ceRNA network frame plays a remarkable role in CRC initiation and progression. Moreover, PVT1 as well as the members of the PVT1-associated crosstalk could be potential targets to inhibit cell proliferation and the invasion of CRC.

### 4.4. CACS19/miR-140-5p/CEMIP

Wang et al. discovered a novel ceRNA interaction of CACS19/miR-140-5p/CEMIP in CRC progression [33]. LncRNA CACS19 is also located on chromosome 8q24.21, which is rich in CRC-related oncogenic lncRNAs. Their study revealed that CACS19 reversed the tumor-suppressive effect of miR-140-5p by competing for the MREs with CEMIP mRNA, miR-140-5p’s direct target, which led to an upregulation of CEMIP mRNA. CEMIP promotes tumor cell survival through the breakdown of glycogen [34]. This investigation uncovered a novel CACS19-related ceRNA mechanism whereby CACS19 enhanced CEMIP by physically harboring miR-140-5p and abolishing its availability, thus contributing to CRC cell proliferation and tumor growth. As a result, all the members of this novel CACS9/miR-140-5p/CEMIP axis could serve as potential therapeutic targets for CRC.

### 4.5. UCA1/miR-28-5p/HOXB3

As an oncogenic lncRNA, UCA1 is upregulated and exerts a tumor-promoting effect in CRC [35,36,37,38]. Cui et al. reported that UCA1 expression is upregulated in CRC and is positively associated with tumor growth and advanced stages [39]. The data showed that the silencing of UCA1 inhibited CRC proliferation and invasion, not only by suppressing MMP-2/9, but also by a novel lncRNA/miRNA interaction. It was proven that UCA1 acted as a ceRNA of the tumor suppressor miR-28-5p via 3′UTR binding and thereby impeded the repression of HOXB3, which promoted cancer cell proliferation and invasion. This study also indicated that both UCT1 knockdown and miR-28-5P overexpression could be strategies for CRC suppression.

### 4.6. Other lncRNA/miRNA/mRNA Axis

Additionally, numerous CRC-associated lncRNA/miRNA/mRNA axis have been reported in recent studies; they are involved in CRC cell proliferation, migration, invasion, tumor growth, and metastasis. Most of the previously identified lncRNAs, located on different chromosomes, exert a regulatory effect as ceRNAs of miRNAs in CRC. For instance, LncRNA H19 promotes CRC development and malignancy transformation, partly through the H19/miR-675/RB axis [40]. LncRNA DANCR exerts its oncogenic function on CRC tumor growth and liver metastasis through the DANCR/miR-577/HSP27 axis [41]. LncRNA ZNF148 induces cell proliferation in CRC via the ZNF148/miR-101,144,336,356/TOP2A axis [42]. LncRNA NEAT1 is proven to be positively associated with CRC tumor differentiation, metastasis, and TNM stages through the NEAT1/miR-495-3p/CDK6 axis [27,43]. These competitive interactions reveal the hidden lncRNA-mediated mechanisms underlying CRC initiation and progression.

## 5. LncRNAs as ceRNA in the EMT and Cell Stemness Formation in CRC

In cancer, epithelial to mesenchymal transition (EMT), marked by the loss of E-cadherin, enables the epithelial cells of a primary tumor to lose cell polarity and break the cellular adhesion constraints, which allows carcinoma cells to gain migratory and invasive properties and be mesenchymal-like towards aggressive malignancy [44,45,46]. As a pathological mechanism, EMT has been reported to initiate CRC metastasis from the primary tumor to distant sites, especially to liver and lymph nodes. Moreover, previous studies have indicated a strong association between the chemoresistance and acquisition of EMT in various cancers [29]. Clinical evidence clearly suggests that the altered expression of EMT-related markers (e.g., the reduced cell adhesion molecules E-cadherin and increased mesenchymal marker, Vimentin [47,48,49]) is not only involved in CRC metastasis, but also in chemotherapy resistance [50,51,52,53,54,55]. Emerging evidence indicates that lncRNAs could be considered as novel EMT markers in various cancers. Mechanically, several lncRNAs promote EMT formation via traditional mechanisms. For instant, lncRNA MALAT1 showed a positive correlation with the expression level of EMT-transcriptional factor, ZEB1, ZEB2, and SNAI2 [56]. HOTAIR mediated EMT formation, together with PRC2 and LSD1 proteins, reprogramming the chromatin profile of the epithelial cells to that of mesenchymal cells [57]. Furthermore, recent studies based on the ceRNA mechanism provide a new understanding of lncRNA-associated EMT formation CRC.

### 5.1. H19

LncRNA H19, located on chromosome 11 in humans, contributes to EMT formation by regulating relevant signaling pathways or molecules in colorectal cancer, lung cancer, breast cancer, and pancreatic cancer [58,59,60,61]. Transforming growth factor-β1 (TGF-β1) is a conventional inducer to establish an EMT model in various epithelial cells, and potentiated H19 expression has been found in TGF-β1-induced EMT model in CRC cells [58,62]. Furthermore, cells expressing H19 displayed mesenchymal-like properties. Recently, H19 has been reported to promote EMT in CRC via a novel ceRNA mechanism, suggesting that upregulated H19 is a biomarker of CRC metastasis and, more importantly, could be a promising therapeutic target for CRC treatment.

#### 5.1.1. H19/miR-138/Vimentin and H19/miR-200a/ZEB1, ZEB2

Liang et al. reported that, in colon cancer cells, upregulated H19 competitively harbors miRNA-138 and miRNA-200a, which hampers the complementary bindings between miRNAs and the mRNAs of Vimentin, ZEB1, and ZEB2. miR-138 and miR-200 are known to suppress EMT in cancer cells by suppressing the translation of EMT regulators, such as Vimentin and ZEB1/2 [63,64,65]. Long et al. detected that the ectopic expression of miR-138 results in a suppression of CRC metastasis by inhibiting the EMT inducer TWIST2 in CRC cells and tissues [66]. Similarly, in breast cancer, miR-138 inhibited EMT by increasing E-cadherin and reducing Vimentin, which is a filament protein highly expressed in mesenchymal cells [67]. Additionally, miR-200a, belonging to the miR-200 family, inhibits colon cancer EMT by targeting and repressing ZEB1/2 [68]. In summary, this study found that H19 promoted EMT formation in CRC through H19/miR-138/Vimentin and H19/miR-200a/ZEB1/2 ceRNA networks [58], by which: 1) H19 acted as a ceRNA to compete for free miR-138 and miR-200a, which sequestered these two miRNA species and freed the 3′UTR of targeted mRNAs; 2) due to the competitive binding events, H19 abolished the suppressive effect on Vimentin and ZEB1/2. Similarly, the investigation of Yang et al. found that H19 promoted the migration and invasion of CRC cells, as well as CRC tumor growth and liver metastasis, by competitively sponging miR-138 to derepress the oncoprotein HMGA1 [69,70].

#### 5.1.2. H19/miR-29-3b/PGRN

Intriguingly, another study conducted by Ding et al. confirmed that H19 promoted EMT in CRC via the ceRNA mechanism [71]. Clinical pathological studies of CRC patients indicated that overexpressed H19 and downregulated miR-29-3b were both observed in the tissue samples of CRC patients with poor differentiation, advanced stages, and distant metastasis. Consistently, H19 attenuated E-cadherin and increased Vimentin and Snail in CRC cells. MiR-29-3b acts as a tumor suppressor, which increased E-cadherin and constrained Vimentin and Snail. More importantly, miR-29-3b suppressed Wnt/β-catenin signaling and c-Myc, Cyclin D1 by inhibiting PGRN (a growth factor involved in tumorigenesis and would healing) [72]. On the other hand, PGRN exhibited the regulatory effect on EMT of epithelial ovarian cancer cells [73]. Based on software prediction and in vitro evidence, it is found that 1) H19 was positively correlated with the pathogenesis of EMT of CRC; 2) H19 competitively sponged miR-29-3b; and 3) H19 negatively regulated miR-29-3b abundance and abolished its suppressive effect on PGRN and Wnt signaling in CRC cells. Overall, H19 promoted EMT in colorectal cancer via a novel ceRNA network of H19/miR-29-3b/PGRN/Wnt signaling axis.

### 5.2. HIF1A-AS2/miR-129-5p/DNMT3A

LncRNA HIF1A-AS2, which is an antisense transcript of HIF1A, plays a key role in the tumorigenesis of various cancers, such as glioblastoma and bladder cancer [74,75,76]. A recent study by Lin et al. reported that HIF1A-AS2 induced the EMT formation of colorectal cancer through the miR-129-5p/DNMT3A ceRNA pathway [77]. Clinical evidence showed a strong correlation between the highly-expressed HIF1A-AS2 and advanced stage in CRC patients. HIF1A-AS2 promoted cell invasion and EMT of CRC cells by inhibiting E-cadherin. It was observed that HIF1A-AS2 enhanced DNMT3A by competitively binding with miR-129-5p and diminishing its suppressive effect on the translation of DNMT3A mRNA. DNMT3A is a member of the DNA methyltransferases (DNMTs) family [78]. The dysregulation of the DNMTs family, including DNMT1, DNMT3A, and DNMT3B, has been reported to be responsible for malignant transformation in colon, lung, liver, and breast cancer [79,80,81,82]. HIF1A-AS2 augmented DNMT3A by acting as a ceRNA of miR-129-5p, leading to EMT formation and CRC progression. This study not only identified the oncogenic function of HIF1A-AS2 in CRC, but also provided new therapeutic targets based on the HIF1A-AS2/miR-129-5p/DNMT3A ceRNA network.

### 5.3. UICLM/miR-215/ZEB2

Chen and colleagues first characterized the role of lncRNA UICLM-mediated ceRNA network in CRC liver metastasis. UICLM promoted EMT formation and cell stemness through the UICLM/miR-215/ZEB2 network [83]. Clinicopathological evidence showed that UICLM is highly overexpressed in CRC patients with liver metastasis, suggesting that UICLM is required for the CRC cell EMT process and stemness formation. In vitro evidence further demonstrated that UICLM enhanced ZEB2, the activator of EMT and liver metastasis, by competitively harboring miR-215, thus antagonizing its inhibitory effect on the translation of ZEB2. This finding provided the missing link between lncRNA and CRC liver metastasis, as well as a novel paradigm for the ceRNA mechanisms underlying CRC metastasis. Taken together, UICLM is a promising therapeutic target since the knockdown of UICLM not only negatively regulates ZEB2, but also markedly inhibits stemness-related genes such as SOX2, Notch1, and cancer stem cell (CSC)-associated surface antigens, such as CD44 and CD24.

## 6. LncRNA as ceRNA in the Inflammation Formation in CRC

Chronic inflammation plays a causal role in CRC development. An increased risk of developing CRC has been observed in patients with inflammatory bowel disease (IBD) [84]. IBD are chronic inflammatory conditions of the gastrointestinal (GI) tract and mainly include Crohn’s disease (CD) and ulcerative colitis (UC) [85]. CD has been acknowledged as transmural intestinal inflammation, which could impact any part of the GI tract, while UC is a superficial inflammation that typically involves the large bowel [86]. CRC developed from IBD is known as colitis-associated colorectal cancer (CAC). LncRNAs are uniquely expressed in certain IBD conditions and play critical roles in the inflammation process in both CD and UC [87]. For instance, lncRNA DQ786243 is the first reported lncRNA that participates in the pathogenesis of CD and CRC; ANRIL (also called CDKN2B-AS1) is dysregulated in an IBD pathology context and may regulate inflammatory responses by acting as a component of the nuclear factor-κB (NF-κB) pathway; the UC-associated lncRNA BC012900 represents its potential as a diagnostic biomarker in IBD progression since BC012900 is particularly differentially expressed in UC samples [88,89,90]. Notably, several lncRNAs are involved in the autophagy process in CRC development [91,92]. Autophagy is a cellular hemostatic process, whereby the cell eliminates intracellular components and removes harmful pathogens from the cytoplasm. Autophagy is critical to inflammation and the tumor microenvironment of CRC since it impacts the hemostasis and survival of inflammatory cells, including macrophages, neutrophils, T lymphocytes, and B lymphocytes [93]. LncRNA KCNQ1OT1 and lincRNA POU3F3 knockdown induced autophagy in CRC cell lines [94,95]. In particular, some lncRNAs regulate autophagy in the tumorigenesis of CRC and CAC by behaving as ceRNA of inflammation/autophagy-related miRNAs.

The study conducted by Zheng et al. found that lncRNA HAGLROS regulated the autophagy and apoptosis of HCT116 cells by acting as ceRNA of autophagy-related miRNA, miR-100 and inhibiting its availability [96]. miR-100 targeted the 3′UTR of autophagy-related 5 (ATG5) and consequently inhibited its expression, thus resulting in the suppression of cell autophagy. Also, miR-100 promoted cell autophagy in osteosarcoma and hepatocellular carcinoma (HCC) [97,98]. Knockdown of HAGLROS efficiently inhibited autophagy and induced apoptosis via the ceRNA network linking autophagy-related miRNA and proteins. In addition, Liu et al. reported that lncRNA UCA1 contributed to the autophagy of CRC cells by harboring miR-185-5p to activate the WNT1-inducible signaling pathway protein 2 (WISP2)/β-catenin pathway [99].

Tian et al. revealed that inflammation-related lncRNA MALAT1 and miR-663a constitute a ceRNA network in CRC cells through sequence-dependent binding [100]. They illustrated that MALAT1 reduced miR-663a expression via a ceRNA mechanism, to prevent the degradation of most of miR-663a’s targets (P53, PIK3CD, P21, CXCR4, TGFB1, and JUND) in CRC cells and tissues. Notably, MALAT1 has been identified as an inflammatory regulator in human systematic lupus erythematosus, and miR-663a is involved in the pathology in chronic inflammation, whereby it induced pro-inflammatory cytokines to promote inflammation in joints [101,102]. MALAT1 and miR-663a may be involved in CRC development and inflammation formation.

## 7. LncRNA as ceRNA in Chemoresistance/Radioresistance of CRC

Chemo-/radioresistance causes cancer recurrence and failed clinical outcome [103]. However, the molecular mechanisms of chemo-/radioresistance remain poorly understood, which prevents the success of cancer treatment. Since studies have indicated that the ceRNA mechanism has strong relevance to cancer initiation and progression, it is increasingly speculated that the lncRNA-mediated ceRNA network plays a key role in the acquirement of chemo-/radioresistance in cancers. The latest research has identified that the lncRNA/miRNA/mRNA pathway regulated doxorubicin-based, 5-fluoroural-based, and oxaliplatin-based chemotherapies for CRC. For example, MALAT1 conferred oxaliplatin resistance to CRC via the miR-218/EZH2 axis by promoting EMT formation, suggesting that lncRNA, as well as members of the lncRNA-related ceRNA crosstalk, could be therapeutic targets for CRC [104].

### 7.1. XIST

Doxorubicin (DOX) is a commonly used chemotherapeutic agent for CRC treatment. However, the occurrence of chemoresistance limits its application in clinic. A previous study has suggested that the acquisition of DOX resistance is related to the EMT formation in CRC cells [105]. After knockdown of TGFβ signaling in CRC cells, the EMT process was reversed and the sensitivity to DOX was significantly increased. Apart from EMT, recent studies revealed that lncRNA XIST participated in the mechanism of DOX resistance acquisition in colorectal cancer. The study conducted by Zhu et al. found that XIST induced DOX resistance by sponging miR-124 to upregulate SGK1 expression [106]. It has been reported that SGK1 could confer chemo- and radioresistance in various malignancies [107]. Amato et al. reported that activated SGK1 induced the DOX resistance in renal carcinoma cells [108]. Considering the involvement of the XIST-mediated ceRNA mechanism in DOX-resistant CRC cells, knockdown/silencing of XIST might be a potential strategy to overcome chemoresistance in human CRC cells. Additionally, Zhang et al. confirmed that XIST promoted CRC progression under the influence of chemo drugs via a novel ceRNA-dependent mechanism. The upregulated XIST competitively interacted with miR-30a-5p at the 3′UTR and, thus, alleviated the repression effect on its downstream target, the mRNA of ROR1 [109]. Previous studies reported that ROR1 functions as a regulator of EMT-related genes and highly expressed ROR1 exhibited a positive correlation to the malignancy attributes of CRC patients [110,111]. Furthermore, they discovered that atractylenolide II, a natural product extracted from the dried rhizome of *Atractylodes macrocephala*, successfully enhanced the sensitivity of CRC cells to several first-line chemo drugs, such as 5-fluorouracil (5-FU), doxorubicin (DOX), and cisplatin, by disturbing the XIST/miR-30-5p/ROR1 ceRNA network [112]. Mechanically, Atractylenolide II suppressed the cell proliferation capacity by decreasing XIST and ROR1 expression, thus efficiently reversing the XIST-induced chemoresistance of CRC cells. In summary, these findings indicated the crucial role of XIST-mediated ceRNA crosstalk in the chemoresistance of CRC. More importantly, they identified the XIST ceRNA axis as a promising target to overcome chemoresistance in CRC.

### 7.2. TUG1

5-fluorouracil (5-FU) is a primary chemotherapy drug for various cancers, particularly for colorectal cancer. 5-FU inhibits the nucleotide synthetic enzyme thymidylate synthase (TYMS) and incorporates its metabolites into DNA and RNA, which leads to the inhibition of the DNA synthesis and the disruption of RNA processing and post-transcriptional modification in cancer cells [113]. However, chemoresistance limits the response rates of 5-FU-based chemotherapy for advanced CRC treatment. A recent study suggested that lncRNA TUG1 modulated 5-FU resistance in CRC cells via the TUG1/miR-197-3p/TYMS signaling [114]. Researchers found that TUG1 promoted the cell viability and proliferation of 5-FU-resistant CRC cells by a novel ceRNA mechanism. TUG1 upregulated TYMS by competitively sequestering miR-197-3p. TYMS is a direct target of miR-197-3p. This interaction decreased the miR-197-3p expression and relieved its suppression effect on TYMS. Moreover, this study reported that TUG1 is positively correlated to 5-FU resistance as well as CRC recurrence. TUG1 could be considered not only as a potential silencing target to overcome 5-FU resistance in CRC cells, but also as a biomarker to assess the clinical response to 5-FU in CRC patients. The TUG1-mediated ceRNA mechanism also plays a key role in methotrexate (MTX) resistance in colorectal cancer. MTX has been used for cancer treatment since 1956 [115]. It works as an inhibitor of the dihydrofolate reductase (DHFR) enzyme to block the biosynthesis of DNA [116]. However, MTX resistance limits its clinical outcomes in CRC therapy. Li et al. first discovered that TUG1 is highly upregulated in MTX-resistant CRC tumors [117]. Based on software predictions, TUG1 and mRNA of CPEB2 are both potential targets of miR-186. Further studies showed that overexpressed TUG1 elevated CPEB2 by competitively hijacking miR-186. This study found that CPEB2 could confer MTX resistance to CRC cells. The role of CPEB2 in CRC remains unknown. Overall, TUG1 exhibited a positive correlation to chemoresistance in CRC cells and tissue specimens via ceRNA networks, and therefore represented a therapeutic target to overcome drug resistance for CRC treatment.

### 7.3. MEG3

Oxaliplatin (OXA) is a first-line platinum drug with proven suppression activity upon colon tumors [118]. OXA resistance caused failure in CRC treatment via an unclear mechanism. Li et al. reported that MEG3 enhanced the susceptibility of CRC to oxaliplatin by promoting cell cytotoxicity [119]. Importantly, Wang et al. identified that overexpressed MEG3 ameliorated CRC sensitivity to oxaliplatin via the ceRNA mechanism [120]. MEG3 functions as ceRNA of miR-141, which results in the inhibition of miR-141 and thus the de-repression of downstream target, PDCD4. Notably, PDCD4 sensitized cancer cells to cisplatin, paclitaxel, and docetaxel by acting as a tumor suppressor to induce apoptosis [121,122,123]. This study proves that the overexpression of MEG3 could be a potential strategy to improve therapeutic outcomes for CRC.

### 7.4. UCA1

Clinicopathological evidence obtained from CRC patients highlighted that the expression of UCA1 was correlated with lymph node metastasis, tumor stage, and poor prognosis [124]. A previous study demonstrated that UCA1 increased cisplatin resistance in bladder cancer cells [125]. Recently, Bian et al. observed that UCA1 induced 5-FU resistance in CRC cells via a ceRNA logic, whereby UCA1 inhibited miR-204-5p expression and its activity through competitively enriching miR-204-5p at its 3′UTR binding sequences, leading to the release of the mRNA of RAB22A and BCL2 [38]. Therefore, as the inducers of cell apoptosis, RAB22A and BCL2 protein level and activities were activated in 5-FU resistant cells. A subsequent study confirmed that knockdown of RAB22A increased chemo responsiveness in CRC cells [126]. This investigation illustrated that UCA1 induced 5-FU resistance via the miR-204-5p/BCL2, RAB22A ceRNA axis.

### 7.5. Linc00152

Emerging evidence revealed that Linc00152 promoted tumorigenesis via ceRNA pathways in various cancers. For example, Linc00152 increased cell proliferation by sponging miR-193b-3p in osteosarcoma, and promoted leukemogenesis by targeting the miR-193a/CDK9 axis [127,128]. A recent study evaluated the pivotal role of linc00152 in the ceRNA mechanism, by which it conferred chemoresistance in colorectal cancer. Yue et al. demonstrated that linc00512 reduced the sensitivity of CRC cells to oxaliplatin via the miR-193a-3p/ERBB4/AKT pathway to regulate the expression of miR-193a-3p and ERBB4 [129]. In CRC, miR-193a-3p showed tumor suppressor properties by arresting the cell cycle and inducing apoptosis; in contrast, ERBB4 enhanced the survival rate of CRC cells with activated P13K, EGFR pathway, and inflammation factors [130,131]. Thus, linc00512 induces resistance in oxaliplatin-treated CRC cells due to its induction effect on ERBB4 by competitively harboring and titrating miR-193a-3p. Another study confirmed that linc00512 was involved in chemoresistance by more than one ceRNA logic [132]. Linc00512 promoted 5-FU resistance in CRC via miR-139-5p/NOTCH1 axis by driving growth and metastasis. Mechanically, overexpressed linc00512 inhibited tumor suppressor miR-139-5p to relieve its repression on NOCH1, which was positively related to the poor survival rate of CRC. Taken together, these studies revealed that the linc00512-mediated ceRNA network is closely correlated with the occurrence of chemoresistance in CRC.

### 7.6. SNHG6

Recent studies elucidated that LncRNA SNHG6 (also known as ncRAN) contributed to 5-FU resistance in CRC by promoting CRC cell autophagy [133]. Autophagy is a cellular self-digestion process that contributes to tumor development and resistance to chemotherapy drugs [134,135,136,137,138]. SNHG6 induced autophagy with enhanced autophagosome formation, thus leading to the acquisition of 5-FU resistance in CRC cells. ULK1 is an important initiator of autophagy. It was observed that SNHG6 interacted with ULK1 and motivated its autophagy-induced function by sequestrating miR-26a-5p, which is an autophagy suppressor. This study illustrated that lncRNAs could regulate autophagy and autophagy-related chemoresistance through the ceRNA network in CRC cells.

### 7.7. CACS15

Gao et al. investigated the molecular mechanism of the oncogenic lncRNA CACS15 in oxaliplatin-resistant CRC cells [139]. They discovered that CASC15 was upregulated in OXA-resistant CRC cells, while, CASC15-silenced OXA-resistance CRC cells regained sensitivity to OXA. Mechanistically, CACS15 promoted oxaliplatin resistance to CRC cells by elevating ABCC1 via the CACS15/miR-145 axis. In this case, downregulation of CASC15 could be a therapeutic strategy to facilitate the response of CRC patients to oxaliplatin.

### 7.8. TINCR

Besides chemotherapy, radiation is a widely used CRC treatment. However, the high occurrence of radioresistance results in failed radiotherapy in CRC patients [140]. Kang et al. demonstrated that knockdown of TINCR inhibited radioresistance via the miR-137/TCF4 ceRNA axis [141]. They speculated that the stemness of CRC cells was associated with the radioresistance since cancer stem cells possess tumor-initiating capacity [142]. Intriguingly, TINCR and TCF4 both induced stemness in radioresistant CRC cells. Though the mechanism is still unknown, this study provided potential biomarkers for assessing the outcomes of radioresistance for CRC therapy.

Collectively, mounting evidence indicated the involvement of lncRNA-mediated ceRNA interplay in chemo-/radioresistance in colorectal cancer. These findings provided potential predictors for the acquisition of chemo-/radioresistance and shed light on the clinical implication of lncRNA-associated ceRNA mechanisms in overcoming cancer chemoresistance. So far, there are still a large number of resistance-related lncRNAs in CRC that have not been studied. For example, lncRNA CCAT1 enhanced the 5-FU resistance in CRC cells by an unidentified mechanism. Therefore, further investigations of lncRNAs in CRC chemoresistance are needed to ensure better clinical outcomes.

## 8. Databases for the Prediction of lncRNA-Associated ceRNA Interactions

Bioinformatics and software algorithms are potent tools for the prediction and validation of RNA interactions based on the ceRNA hypothesis. Below we list some freely available databases for the exploration of lncRNA-associated ceRNA networks.

starBase v2.0 (http://starbase.sysu.edu.cn/) is a database used for the systematic identification of miRNA‒ncRNA (including lncRNAs, pseudogenes, circRNAs) and miRNA‒mRNA interaction networks from 108 CLIP-Seq (HITS-CLIP, PAR-CLIP, iCLIP, CLASH) datasets. More importantly, starBase v2.0 is the first database containing miRNA‒pseudogene and miRNA‒circular RNA interactions based on Ago and RBP binding sites, which contributes to the construction of miRNA‒lncRNA ceRNA regulatory networks [143].

DIANA-LncBase (www.microrna.gr/LncBase) is a comprehensive annotation first established for the transcriptome-wide prediction of lncRNA and miRNA functional interactions based on MREs in human and mouse lncRNAs. It also details information for each miRNA‒lncRNA pair, such as representation of the binding sites, MREs conservation, and prediction scores, as well as lncRNA tissue expression [144].

spongeScan (http://spongescan.rc.ufl.edu) is the first web algorithm used for the identification of putative MREs in lncRNAs and the assessment of their likely behavior as ceRNAs. This novel application is based on sequence complementarity and sequence information is available for any organism [145].

lnCeDB database (http://gyanxet-beta.com/lncedb/) was developed to browse for lncRNA‒mRNA pairs sharing common miRNA, and to estimate the probability of each pair being ceRNAs in 22 human tissues [146].

LncNetP is a large-scale RNA-seq database developed for the systematic identification and prioritization of disease/cancer-associated lncRNAs based on the ceRNA hypothesis [147].

Other databases: The SomamiR database (http://compbio.uthsc.edu/SomamiR) was designed for detecting potential alterations of miRNA‒ceRNA interactions in cancer somatic mutations, including miRNA‒lncRNA interplay [148]. miRsponge was developed from the data on 500 miRNA sponge interactions and 463 ceRNA relationships from 11 species based on published articles [149]. PceRBase (http://bis.zju.edu.cn/pcernadb/index.jsp) is a plant ceRNA database containing potential ceRNA pairs from 26 plant species [150]. Linc2GO is the first lincRNA functional annotation database based on the ceRNA hypothesis, and is freely available (http://www.bioinfo.tsinghua.edu.cn/~liuke/Linc2GO/index.htm) [151]. 

## 9. Conclusions and Future Outlook

While only ~1.5% of the human genome encodes protein-coding genes, the rest of the genome contains noncoding sequences. Most of these non-protein-coding DNA sequences are transcribed into RNA; among those are thousands of miRNAs and tens of thousands of lnRNAs. As discussed in this review, many lnRNAs compete with specific mRNAs in their binding to miRNAs. These lnRNA‒miRNA‒mRNA competitive endogenous RNA networks form a complex and highly regulated mechanism to control gene expression and cellular functions. Since the lncRNA members of the ceRNA networks are frequently involved in the advanced stages of CRC (for example, CACS15, CYTOR, HOTAIR, MALAT1, TUG1, NEAT1 and MIR17HG) these lncRNAs may serve as potent prognostic biomarkers. More importantly, knockdown/overexpression of these abovementioned members within CRC-associated ceRNA networks significantly suppressed CRC progression, suggesting their potential role as therapeutic targets. Despite intensive research on the molecular mechanisms behind colorectal cancer in recent decades, this cancer remains one of the most lethal malignancies due to the frequent occurrence of EMT, metastasis, and chemo-/radioresistance. Research on the novel lncRNA-associated ceRNA network may open up new avenues in CRC therapy and overcoming drug resistance. Our understanding of these ceRNA networks is still in the early stages. The precise mechanisms of their involvement in cancer progression are still largely unknown. More advanced genome-wide algorithms are needed for ceRNA prediction in order to identify new prognostic biomarkers. New strategies need to be developed (such as new delivery methods to deploy siRNA or CRISPR) to target these ceRNA networks for cancer therapy.

## Figures and Tables

**Table 1:** LncRNA/miRNA/mRNA ceRNA network in CRC.

LncRNA	Chromosome Location	Competitor mRNA	Shared miRNA	ceRNA Network	ceRNA Role	Related CRC Hallmark	Ref.
BCAR4	16p13.13	STAT3	miR-665	BCAR4/miR-655/STAT3	Oncogenic	Proliferation, migration	[152]
CACS15	6p22.3	LGR5	miR-4310	CACS15/miR-4310/LGR5	Oncogenic	Proliferation, invasion, TNM stage, metastasis	[153]
CASC19	8q24.21	CEMIP	miR-140-5p	CASC19/miR-140-5p/CEMIP	Oncogenic	Proliferation, invasion, migration, apoptosis, EMT	[33]
CASC2	10q26.11	PIAS3	miR-18a	CASC2/miR-18a/PIAS3/STAT3	Tumor suppressive	Proliferation, tumor growth, G0/G1-S phase transition	[154]
CCAT2	8q24.21		miR-145	CCAT2/miR-145/miR-21	Oncogenic	CSC proliferation and differentiation	[155]
CYTOR	2p11.2	MACC1	miR-3679-5p	CYTOR/miR-3679-5p/MACC1	Oncogenic	TNM stage, perineural and venous invasions	[156]
ENSG00000-231881	6	VEGFC	miR-133b	ENSG00000231881/miR-133b/VEGFC	Oncogenic	Metastasis	[157]
FER1L4	20q11.22		miR-106a-5p	FER1L4/miR-106a-5p	Tumor suppressive	Proliferation, cell cycle	[158]
FOXD2-AS1	1p33	CDC42	miR-185-5p	FOXD2-AS1/miR-185-5p/CDC42	Oncogenic	Proliferation, migration, invasion	[159]
FOXD3-AS1	1p31.3	SIRT1	miR-135a-5p	FOXD3-AS1/miR-135a-5p/SIRT1	Oncogenic	Proliferation, migration, invasion, cell cycle, apoptosis	[160]
GACAT3	2p24.3	SP1, STAT3	miR-149	GACAT3/miR-149/SP1/STAT3	Oncogenic	Proliferation, invasion, migration	[161]
GAS5	1q25.1	PTEN	miR-222-3p	GAS5/miR-222-3p/PTEN	Tumor suppressive	Proliferation, migration, apoptosis	[162]
H19	11p15.5	Vimentin, ZEB1, ZEB2	miR-138, miR-200a	H19/miR-138/Vimentin, H19/miR-200a/ZEB1, H19/miR-200a/ZEB2	Oncogenic	EMT progression	[58]
HAND2-AS1	4q34.1	KLF14	miR-1275	HAND2-AS1/miR-1275/KLF14	Tumor suppressive	Proliferation, invasion	[163]
HOTAIR	12q13.13		miR-34a	HOTAIR/miR-34a	Oncogenic	Metastasis	[164]
HULC	6p24.3	RTKN	miR-613	HULC/miR-613/RTKN	Oncogenic	Proliferation, metastasis	[165]
LINC00460	13q33.2	LIMK2	miR-939-5p	LINC00460/LIMK2/miR-939-5p	Oncogenic	Metastasis	[166]
LINC00668	18p11.31	USP47	miR-188–5p	LINC000668/miR-188-5p/USP47	Oncogenic	Proliferation, metastasis	[167]
LINC00858	10q23.1	YWHAZ	miR-22-3p	LINC00858/miR-22-3p/YWHAZ	Oncogenic	Proliferation, migration, invasion	[168]
LINC01234	12q24.13	SHMT2	miR-642a-5p	LINC01234/miR-642a-5p/SHMT2	Oncogenic	Proliferation	[169]
LINC01296	14q11.2	PDCD4	miR-21a	LINC01296/miR-21a/PDCD4	Oncogenic	Proliferation	[170]
LINC02418	12q24.33	MELK	miR-1273g-3p	LINC02418/miR-1273g-3p/MELK	Oncogenic	Proliferation, apoptosis	[171]
MALAT1	11q13.1	p53	miR-663a	MALAT1/miR-663a/p53	Oncogenic	Proliferation, migration, invasion, apoptosis	[100]
MBNL1-AS1	3q25.1	MYL9	miR-412-3p	MBNL-AS1/miR-412-3p/MYL9	Tumor suppressive	Proliferation, invasion	[172]
MIAT	22q12.1	Derlin-1	miR-132	MIAT/miR-132/Derlin-1	Oncogenic	Tumor growth, metastasis	[173]
MIR17HG	13q31.3	Wnt, β-catenin	miR-17, miR-18a	MIR17HG-miR-17/18a-Wnt/ β-catenin	Oncogenic	Lymph node metastasis, TNM stage	[174]
MNX1-AS1	7q36.3	SEC61A1	miR-218-5p	MNX1-AS1/miR-218-5p/ SEC61A1	Oncogenic	progression of colon adenocarcinoma	[175]
NEAT1	11q13.1	CDK6	miR-495-3p	NEAT1/miR-495-3p/CDK6	Oncogenic	Proliferation, migration, invasion	[43]
OECC	8q24	NF-κB, p38MAPK	miR-143-3p	OECC/miR-143-3p/NF-κB/ p38 MAPK	Oncogenic	Proliferation, apoptosis, migration	[176]
PART-1	5q12.1	DNMT3A	miR-143	PART-1/miR-143/DNMT3A	Oncogenic	Proliferation, metastasis	[177]
PVT1	8q24.21	RUNX2	miR-30d-5p	PVT1/miR-30d-5p/RUNX2	Oncogenic	Proliferation, metastasis	[26]
PVT1	8q24.21	RUNX2	miR-455	PVT1/miR-455/RUNX2	Oncogenic	Proliferation, migration, invasion, apoptosis	[31]
PVT1-214	8q24.21	Lin28	miR-128	PVT1-214/miR-128/Lin28/let-7	Oncogenic	Proliferation, invasion	[32]
ROR	18q21.31		miR-145	ROR/miR-145	Oncogenic	Proliferation, migration, invasion	[178]
SNHG15	7p13	SIRT1	miR-141	SNHG15/miR-141/SIRT1	Oncogenic	Proliferation, apoptosis	[179]
SNHG16	17q25.1	AKT	miR-302a-3p	SNHG16/miR-302a-3p/AKT	Oncogenic	Proliferation	[180]
TP73-AS1	1p36.32	PTEN	miR-103	TP73-AS1/miR-103/PTEN	Tumor suppressive	Proliferation	[181]
TP73-AS1	1p36.32	TGF-a	miR-194	TP73-AS1/miR-194/TGF-a	Oncogenic	Proliferation, migration, invasion	[182]
TUG1	22q12.2	KIAA1199	miR-600	TUG1/miR-600/KIAA1199	Oncogenic	Metastasis, EMT	[183]
TUSC7	3q13.31	CDK6	miR-211-3p	TUSC7/miR-211-3p/CDK6	Tumor suppressive	Proliferation	[184]
UCA1	19p13.12	HOXB3	miR-28-5p	UCA1/miR-28-5p/HOXB3	Oncogenic	Proliferation, migration	[39]
UCC	7p15.2	KRAS	miR-143	UCC/miR-143/KRAS	Oncogenic	Cell growth, invasion	[185]
ucoo2kmd.1	17q11.2	CD44	miR-211-3p	ucoo2lmd.1/miR-211-3p/CD44	Oncogenic	Proliferation	[186]
ZDHHC8P1	22q11.23		miR-34a	ZDHHC8P1/miRNA-34a	Oncogenic	Proliferation, metastasis	[187]
ZFAS1	20q13.13		miR-7-5p	ZFAS1/miR-7-5p	Oncogenic	Proliferation, migration, invasion, apoptosis	[188]
ZFAS1	20q13.13	CDK1/ cyclinB1, p53	miR-590-3p	ZFAS1/miR-590-3p	Oncogenic	Apoptosis, p53 dependent cell cycle control	[189]
ZNFX1-AS1	20q13.13	EZH2	miR-144	ZNFX1-AS1/miR-144/EZH2	Oncogenic	Proliferation, migration, invasion, metastasis	[190]

CRC: colorectal cancer, EMT: epithelial mesenchymal transition, CSC: cancer stem cell, TMN stage: classification of malignant tumors, T (tumor), N (lymph nodes), M (metastasis).

## Abbreviations

ABCC1	ATP Binding Cassette Subfamily C Member 1
AKT	AKT Serine/Threonine Kinase 1
BCAR4	Breast Cancer Anti-Estrogen Resistance 4
BCL2	B-Cell CLL/Lymphoma 2
CACS15	Cancer Susceptibility 15
CASC19	Cancer Susceptibility 19
CASC2	Cancer Susceptibility 2
CCAT1	Colon Cancer Associated Transcript 1
CCAT2	Colon Cancer Associated Transcript 2
CDC42	Cell Division Cycle 42
CDK1	Cyclin-Dependent Kinase 1
CDK6	Cyclin-Dependent Kinase 6
CDK9	Cyclin-Dependent Kinase 9
CEMIP	Cell Migration Inducing Hyaluronidase 1
CPEB2	Cytoplasmic Polyadenylation Element Binding Protein 2
CRNDE	colorectal neoplasia differentially expressed
CXCR4	C-X-C Motif Chemokine Receptor 4
CYTOR	Long Intergenic Non-Protein Coding RNA 152
DANCR	Differentiation antagonizing non-protein noding RNA
DNMT3A	DNA Methyltransferase 3 Alpha
ERBB2	Erb-B2 Receptor Tyrosine Kinase 4
EZH2	Enhancer of Zeste 2 Polycomb Repressive Complex 2
FOXD2-AS1	FOXD2 Antisense RNA 1
FOXD3-AS1	FOXD3 Antisense RNA 1
GACAT3	Gastric Cancer Associated Transcript 3
HAND2-AS1	HAND2 Antisense RNA 1
HAGLROS	HAGLR opposite strand lncRNA
HIF1A-AS2	HIF1A Antisense RNA 2
HOTAIR	HOX Transcript Antisense RNA
HSP27	Heat Shock Protein 27
HULC	Hepatocellular Carcinoma Associated Transcript 1
KCNQ1OT1	KCNQ1 opposite strand/antisense transcript 1
KIAA1199	Cell Migration Inducing Hyaluronidase 1
KLF14	Kruppel-Like Factor 14
KRAS	K-Ras
LGR5	Leucine Rich Repeat Containing G Protein-Coupled Receptor 5
LIMK2	LIM Domain Kinase 2
LINC00460	Long Intergenic Non-Protein Coding RNA 460
LINC00668	Long Intergenic Non-Protein Coding RNA 668
LINC00858	Long Intergenic Non-Protein Coding RNA 858
LINC01234	Long Intergenic Non-Protein Coding RNA 1234
LINC01296	Long Intergenic Non-Protein Coding RNA 1296
LINC02418	Long Intergenic Non-Protein Coding RNA 2418
LSD1	Lysine Demethylase 1A
MACC1	MET Transcriptional Regulator MACC1
MALAT1	Metastasis Associated Lung Adenocarcinoma Transcript 1
MBNL1-AS1	MBNL1 Antisense RNA 1
MEG3	Maternally Expressed 3
MELK	Maternal Embryonic Leucine Zipper Kinase
MIAT	Myocardial Infarction Associated Transcript
MIR17HG	MIR17 Host Gene
MNX1-AS1	MNX1 Antisense RNA 1
MYL9	Myosin Light Chain 9
NEAT1	Nuclear Paraspeckle Assembly Transcript 1
OECC	Overexpressed in Colorectal Cancer lncRNA
PART-1	Prostate Androgen-Regulated Transcript 1
PCAT1	prostate cancer associated transcript 1
PDCD4	Programmed Cell Death 4
PDCD4	Programmed Cell Death 4
PGRN	Progranulin
PIAS3	Protein Inhibitor Of Activated STAT 3
PIK3CD	Phosphatidylinositol-4,5-Bisphosphate 3-Kinase Catalytic Subunit Delta
POU3F3	POU class 3 homeobox 3
POU5F1P1	POU class 5 homeobox 1B
PRC2	Polycomb Repressive Complex 2
PTEN	Phosphatase And Tensin Homolog
PTEN	Phosphatase And Tensin Homolog
PVT1	Plasmacytoma Variant Translocation 1
RAB22A	Ras-Related Protein Rab-22A
ROR	Long Intergenic Non-Protein Coding RNA, Regulator Of Reprogramming
ROR1	Receptor Tyrosine Kinase Like Orphan Receptor 1
RTKN	Rhotekin
RUNX2	Runt-Related Transcription Factor 2
SEC61A1	SEC61 Translocon Alpha 1 Subunit
SHMT2	Serine Hydroxymethyltransferase 2
SIRT1	Sirtuin 1
SNHG15	Small Nucleolar RNA Host Gene 15
SNHG16	Small Nucleolar RNA Host Gene 16
SNRHG6	Small Nucleolar RNA Host Gene 6
SP1	Sp1 Transcription Factor
STAT3	Signal Transducer And Activator Of Transcription 3
TCF4	Transcription Factor 4
TGF-a	Transforming Growth Factor Alpha
TGFB1	Transforming Growth Factor Beta 1
TINCR	Tissue Differentiation-Inducing Non-Protein Coding RNA
TOP2A	Topoisomerase (DNA) II Alpha
TP73-AS1	lncRNA P73 antisense RNA 1T
TUG1	Taurine Up-Regulated 1
TUSC7	Tumor Suppressor Candidate 7
TYMS	Thymidylate Synthetase
UCA1	Urothelial Cancer Associated 1
UCC	A novel lincRNA termed upregulated in CRC
ULK1	Unc-51 Like Autophagy Activating Kinase 1
USP47	Ubiquitin Specific Peptidase 47
VEGFC	Vascular Endothelial Growth Factor C
XIST	X Inactive Specific Transcript
YWHAZ	Tyrosine 3-Monooxygenase/Tryptophan 5-Monooxygenase Activation Protein Zeta
ZDHHC8P1	Zinc Finger DHHC-Type Containing 8 Pseudogene 1
ZEB1	Zinc Finger E-Box Binding Homeobox 1
ZEB2	Zinc Finger E-Box Binding Homeobox 2
ZFAS1	ZNFX1 Antisense RNA 1
ZNF148	Zinc Finger Protein 148
ZNFX1-AS1	ZNFX1 Antisense RNA 1
